# Host Defense Antimicrobial Peptides (HDPs) as Regulators of Hemostasis and Vascular Biology

**DOI:** 10.3390/biom16020220

**Published:** 2026-02-02

**Authors:** Sergio Roberto Aguilar-Ruiz, Francisco Javier Sánchez-Peña, Héctor Maximino Rodríguez-Magadán, Miguel Angel Domínguez-Martínez, Héctor Ulises Bernardino-Hernández, Alba Soledad Aquino-Domínguez

**Affiliations:** 1 Departamento de Biomedicina Experimental, Facultad de Medicina y Cirugía de la Universidad Autónoma Benito Juárez de Oaxaca, Oaxaca de Juárez 68120, Mexico; sarbiomedicina@gmail.com (S.R.A.-R.); sanchezpenafranciscojavier90@gmail.com (F.J.S.-P.); 2 Facultad de Medicina Veterinaria y Zootecnia, Universidad Autónoma Benito Juárez de Oaxaca, Oaxaca de Juárez 68120, Mexico; rodriguezmagadanhectormaximino@gmail.com; 3 Laboratorio de Genética Molecular y Zoonosis, Facultad de Medicina Veterinaria y Zootecnia, Universidad Autónoma Benito Juárez de Oaxaca, Oaxaca de Juárez 68120, Mexico; mdominguez.cat@uabjo.mx; 4 Facultad de Ciencias Químicas, Universidad Autónoma Benito Juárez de Oaxaca, Oaxaca de Juárez 68120, Mexico; hubh.1972@gmail.com; 5 Licenciatura en Nutrición, Universidad Autónoma Benito Juárez de Oaxaca, Oaxaca de Juárez 68120, Mexico

**Keywords:** host defense peptides (HDPs), LL-37, defensins, platelets, immunothrombosis, vascular biology

## Abstract

Host defense peptides (HDPs), ancestral effectors of innate immunity, have emerged as pleiotropic regulators transcending their antimicrobial origins. This review critically examines the complex interplay among HDPs, hemostasis, and tissue repair. We analyze molecular mechanisms governing interactions with platelets and endothelial cells, highlighting a fundamental paradigm shift: platelets and megakaryocytes are active synthesizers of a specific peptide repertoire rather than passive carriers. Functional dualities are elucidated, contrasting LL-37-driven platelet agonism via glycoprotein VI (GPVI) against the amyloid-like stabilization of fibrin by defensins. Based on these mechanisms, we propose a framework wherein HDPs function as concentration-dependent molecular switches between physiological repair and pathological thromboinflammation. Furthermore, the review addresses the hypothesis of “adaptive thrombopoiesis,” where systemic peptide surges act as danger signals to reprogram the function of newly formed platelets. Finally, therapeutic implications are evaluated, emphasizing the design of protease-resistant peptidomimetics to harness protective effects while mitigating vascular toxicity.

## 1. Introduction

Antimicrobial peptides (AMPs), or host defense peptides (HDPs) in mammals, are fundamental components of innate immunity conserved from prokaryotes to humans. In humans, HDPs are classified into two main families: cathelicidins and defensins. LL-37 is the sole human cathelicidin, forming an α-helical structure, and is expressed by neutrophils and epithelial cells. Defensins are cysteine-rich peptides with a triple-stranded β-sheet core, subdivided into α-defensins (HNPs 1–4), primarily stored in neutrophil granules, and β-defensins (hBD-1 to -4), expressed by epithelial cells and actively synthesized by platelets. The versatility of these structural classes is driven by their cationic and amphipathic nature [1,2,3]. HDPs act through a sequence of electrostatic interactions with microbial surfaces, followed by hydrophobic insertion into lipid bilayers, leading to membrane disruption or pore formation. Beyond direct microbicidal activity, HDPs exert a protective role by antagonizing pro-inflammatory bacterial products. They neutralize Lipopolysaccharide (LPS), preventing its interaction with host immune receptors and mitigating systemic inflammation. These peptides can translocate into the cytoplasm to modulate targets such as nucleic acid stability and protein synthesis. Unlike traditional antibiotics, which typically rely on a single molecular target, the multimodal action of HDPs effectively circumvents resistance pathways and eradicates persistent infections [4,5].

**Figure 1:**
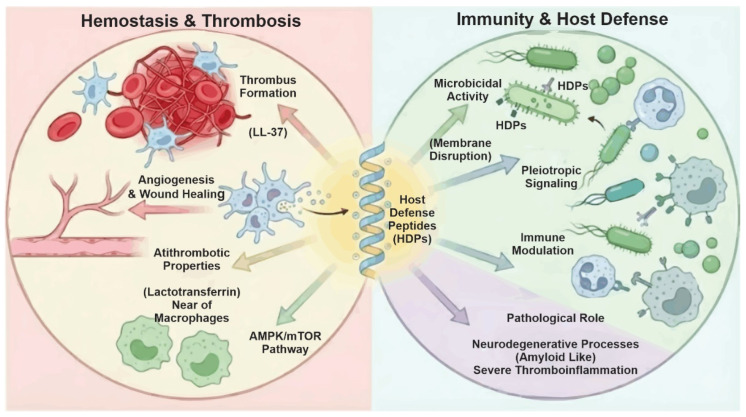
Dual and context-dependent functions of host defense peptides (HDPs) in immunity and hemostasis. They exert direct microbicidal activity and modulate the immune response through receptor-dependent signaling, while also regulating platelet activation, thrombus formation, angiogenesis, and wound healing. These effects are determined by peptide structure, concentration, and microenvironmental context. Dysregulation of HDP activity contributes to pathological thromboinflammation and amyloid-like processes.

Although HDPs are primarily recognized for pathogen neutralization, engineering them for stability and specificity addresses current pharmacokinetic and delivery challenges. A recent paradigm shift has redefined HDPs as pleiotropic regulators rather than simple microbicides [6,7]. Specifically, they modulate key platelet functions, influencing activation and immune responses while reducing aggregation, underscoring their potential for non-canonical therapeutic applications [8,9] (Figure 1).

Paradoxically, HDPs can also mediate pathological processes. Their structural resemblance to amyloid peptides (AMY) enables cross-propagation, linking microbial defense to neurodegeneration [10]. This duality is evident in vascular biology: while cathelicidin LL-37 promotes thrombus formation, lactotransferrin exhibits antithrombotic properties by modulating macrophage autophagy via the AMPK/mTOR pathway [11,12,13] (Figure 1). Rather than simple switches, HDPs are context-dependent regulators; for instance, they often exhibit harmful effects at concentrations of 1–10 μM or under extreme pH levels (pH <6.5 or >8.0) [14,15]. Their role can even be negative during systemic inflammation, contributing to severe thromboinflammatory states [16].

Evidence indicates a coevolution between antimicrobial defense mechanisms and hemostasis, which can be understood through a chronological narrative. This evolutionary journey began in primitive organisms, where basic coagulation mechanisms likely served as the earliest forms of antimicrobial defense. Over time, these mechanisms evolved into more complex systems known as immunothrombosis [17]. Platelets store and release HDPs upon activation, while coagulation factors like fibrinogen can be cleaved into antimicrobial peptides [18,19]. However, this integrated response can become pathological in conditions like sepsis. Furthermore, circulating HDPs may act as systemic “danger signals” in the bone marrow, influencing megakaryopoiesis to produce primed platelets [20]. This review addresses the critical gap in understanding HDPs as dual regulators of immunity and hemostasis.

This review synthesizes current research and proposes an integrated framework in which these peptides act as regulators across these domains. The analysis also discusses ongoing controversies and knowledge gaps. Exploration of the signaling pathways that influence HDPs interactions with bone marrow to understand their role in megakaryopoiesis and platelet priming. These studies will provide a clearer understanding of HDPs’ functionality and help develop targeted interventions in the clinical setting.

## 2. Modulation of Platelet Function by Antimicrobial Peptides and Associated Functional Consequences

HDPs act as autocrine and paracrine signaling molecules, modulating both platelet activation and immune responses. Platelet-derived HDPs are stored within platelet granules or acquired from megakaryocytes, directly influencing platelet function. In contrast, plasma-derived HDPs circulate in the bloodstream and affect platelet behavior through extracellular interactions. This distinction in HDP origin underscores the complex mechanisms by which these peptides can either promote pro-thrombotic and immune-primed states or attenuate platelet activation to prevent pathological occlusion. These pathways demonstrate how varying physiological and pathological contexts regulate platelet function via HDPs [21,22].

LL-37 is stored in α-granules and released upon platelet activation by thrombin or collagen. Following release, LL-37 acts as a potent platelet activator via mechanisms distinct from those of classical hemostasis [11,23]. This mechanism sensitizes platelets to sub-threshold concentrations of conventional agonists. This mechanism is especially relevant in sepsis, where systemic LL-37 lowers the activation threshold and speeds platelet recruitment to neutrophil extracellular traps (NETs), increasing the risk of disseminated intravascular coagulation (DIC). The pro-thrombotic effect of LL-37 is substantial, as it activates Src kinase and Akt (Ser473) signaling pathways to drive thrombo-inflammatory responses. The degree of activation depends on the local peptide concentration [11,16,24].

## 3. Structure–Activity Relationship for Canonical and Non-Canonical Functions

Understanding the structure–activity relationship of HDPs is key to designing safer antimicrobial peptide analogs for future therapeutic use. While the canonical function, direct lysis of microbial membranes, depends on global biophysical properties like net charge and hydrophobicity, recent evidence shows that more subtle and specific structural factors govern non-canonical functions such as immunomodulation, angiogenesis, and hemostasis regulation [25]. The shift between a microbicidal and a signaling state is finely regulated by concentration gradients, oligomerization states, and post-translational modifications such as citrullination, which can drastically alter affinity for cellular or tissue targets and their effects [26,27,28]. Two families of antimicrobial peptides predominate: the α-helical cathelicidins and the β-laminar defensins [29,30]. The potential crosstalk between cathelicidins and defensins may inform future biomaterial design, offering new opportunities for therapeutic innovation.

### 3.1. LL-37 Structural Plasticity and Its Functional Implications in Immune and Hemostatic Regulation

LL-37, the only human member of the cathelicidin family, demonstrates unique structural plasticity that underpins its dual role in immunity and hemostasis [8,31,32]. It is released from the precursor hCAP18 by Proteinase 3 during neutrophil degranulation or by the kallikrein network (KLK5/7) in cutaneous barriers [33,34]. In aqueous solution, LL-37 adopts a disordered random coil conformation. However, upon exposure to physiological saline or lipid interfaces, it rapidly transitions to a defined α-helical structure. This conformational shift facilitates self-assembly into dimers, tetramers, and supramolecular fibers, a process confirmed in solution by techniques such as Circular Dichroism and Size-Exclusion Chromatography. While solid-state NMR and crystallography provide atomic-level details of these structures, solution-phase oligomerization is the biologically relevant state that functions as a selectivity filter: monomers are favored for bacterial membrane insertion, whereas oligomers are required to engage and activate host surface receptors like GPVI [35,36,37,38].

The amphipathic nature of the LL-37 helix (residues 1–37: LLGDFFRKSKEKIGKEFKRIVQRIKDFLRNLVPRTES) is illustrated by the clustering of hydrophobic residues (Leu, Phe, Ile, Val) on one face and hydrophilic and cationic residues (Lys, Arg) on the opposite face [39,40]. This spatial arrangement enables the snorkel effect, in which the long side chains of lysine and arginine interact with lipid phosphate headgroups [41]. This interaction stabilizes the peptide at the membrane interface and allows insertion of its hydrophobic core into the acyl chain region [42]. Clinical evidence indicates that this structural shift is required to activate surface receptors such as GPVI on platelets and particularly relevant in diseases such as psoriasis, where increased LL-37 levels are observed in lesions, and cystic fibrosis, where LL-37’s interaction with neutrophil elastase in sputum contributes to airway inflammation [39,40,41,42,43,44].

### 3.2. Structural Motifs Dictate the Hemostatic Activity of Host Defense Peptides

Structural motifs drive HDP functional specificity by mediating binding to distinct host receptors. Specifically, the amphipathic α-helical conformation of LL-37 facilitates its binding to the G protein-coupled formyl peptide receptor 2 (FPR2), also known as the Lipoxin A4 Receptor (ALX). This FPR2/ALX interaction triggers rapid platelet activation [11].

Nevertheless, pharmacological antagonism of FPR with WRW4, or its genetic ablation in murine models, indicates that FPR2 does not fully account for all LL-37-induced platelet reactivity. This residual activation suggested the involvement of secondary receptors, leading to the identification of GPVI—the primary collagen receptor—as a critical functional target for this peptide [11,32].

**Figure 2:**
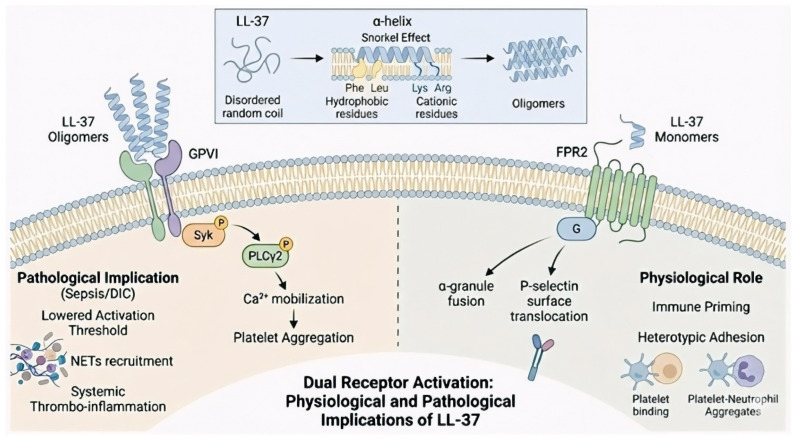
Structural plasticity and dual receptor signaling of LL-37 in platelets. The upper panel illustrates the conformational transition of LL-37 from a disordered coil to an amphipathic α-helix, highlighting the “snorkel effect,” followed by peptide oligomerization. The lower panel depicts divergent functional outcomes depending on the structural state of LL-37. Oligomeric LL-37 activates the glycoprotein VI (GPVI)/spleen tyrosine kinase (Syk)/phospholipase C gamma 2 (GPVI/Syk/PLCγ2) signaling axis, leading to intracellular Ca^2+^ mobilization and platelet aggregation, thereby promoting pathological thrombo-inflammation in sepsis and disseminated intravascular coagulation. In contrast, monomeric LL-37 supports physiological immune priming and heterotypic cell adhesion through formyl peptide receptor 2 (FPR2)-mediated α-granule translocation of P-selectin.

A prominent example of this structural selectivity is the direct interaction of LL-37 with GPVI, the primary collagen receptor. This interaction is substantiated by two complementary lines of biochemical evidence: first, functional mimicry, where LL-37 triggers a tyrosine phosphorylation cascade involving the Fc receptor gamma-chain (FcRγ), spleen tyrosine kinase (Syk), and phospholipase C gamma 2 (PLCγ2) that is identical to the pattern induced by canonical ligands such as collagen or collagen-related peptide (CRP); and second, competitive inhibition, demonstrated by a mutual blockade where pre-incubation with LL-37 prevents CRP binding, while saturation of the collagen-binding site on GPVI abrogates LL-37-mediated platelet activation [30] (Figure 2).

Complementing these signaling pathways, LL-37 modulates the platelet phenotype by mobilizing α-granules through FPR2. Flow cytometric analysis demonstrates that LL-37 induces a dose-dependent (10–20 µM) surface translocation of P-selectin (CD62P), a process significantly attenuated by selective FPR2 blockade. This upregulation is central to a broader activation repertoire that concurrently elevates immune sensors, including Toll-like receptors 2 and 4 (TLR-2, TLR-4), and Lectin-like oxidized low-density lipoprotein receptor-1 (LOX-1), as well as integrins such as CD41 (αIIbβ3). Consequently, this P-selectin-enriched surface primes platelets for heterotypic adhesion, effectively bridging hemostatic functions with innate immune responses, augments thrombus formation, and facilitates platelet-neutrophil aggregate formation [9,11]. Although LL-37 clearly induces CD62P exposure, its precise mechanism of action remains unresolved. Current hypotheses converge on two possibilities: a receptor-mediated biochemical pathway (LL-37 engages FPR2, leading to regulated degranulation) and a biophysical membrane-perturbation mechanism (peptide disrupts the platelet membrane, facilitates Ca^2+^ influx, and induces granule release). While these models differ fundamentally—with the receptor-mediated pathway being pharmacologically targetable and the biophysical model governed by concentration-dependent properties—the functional outcome is consistent: LL-37 drives platelets into a state of heightened immune vigilance.

The structure–activity relationship of HDPs provides a critical mechanistic explanation for this receptor selectivity [32]. Beyond the net cationicity—which facilitates the initial electrostatic attraction to the anionic platelet glycocalyx—the precise spatial arrangement of charged and hydrophobic residues constitutes a distinct structural motif, functioning as a molecular ‘barcode’ explicitly read by receptors such as FPR2 and GPVI [30]. Consequently, endogenous antimicrobial peptides like LL-37 act as potent functional agonists: they actively engage these receptors to trigger signaling cascades, granule mobilization, and the surface upregulation of CD62P and activated αIIbβ3 [8]. This agonist mechanism stands in sharp pharmacological contrast to the mode of action of exogenous antithrombotic toxins, specifically snake venom disintegrins. Unlike LL-37, disintegrins present a rigid loop containing an RGD (Arg-Gly-Asp) motif structurally optimized to dock into the ligand-binding pocket of integrin αIIbβ3, thereby acting as high-affinity competitive antagonists that sterically block fibrinogen binding and inhibit aggregation [45,46].

By situating LL-37 within this pharmacological framework, its capacity to selectively drive an immunothrombotic phenotype without compromising physiological availability is realized. This mechanism is especially relevant in sepsis, where systemic LL-37 lowers the activation threshold and speeds platelet recruitment to NETs, increasing the risk of DIC. The pro-thrombotic effect of LL-37 is substantial, as it activates Src kinase and Akt (Ser473) signaling pathways to drive thrombo-inflammatory responses. The degree of activation depends on the local peptide concentration [11,16,24].

### 3.3. Conformational Control of Immunothrombosis: Rigid Arrays vs. Proteolytic Regulators

**Figure 3:**
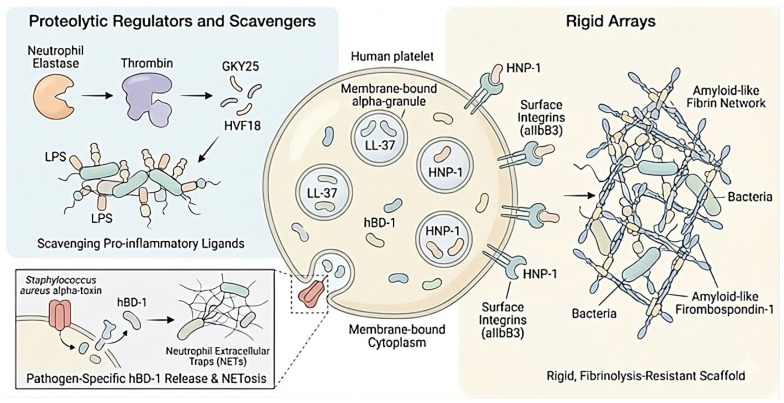
Conformational control of immunothrombosis: rigid arrays versus proteolytic regulators. Human platelets serve as a central reservoir of HDPs, including human neutrophil peptide-1 (HNP-1), LL-37, and human β-defensin-1 (hBD-1). Rigid arrays (right panel): HNP-1 modulates platelet surface integrins, particularly αIIbβ3, and promotes the precipitation of fibrinogen and thrombospondin-1 into amyloid-like fibrillar networks. These rigid structures generate a fibrinolysis-resistant scaffold that facilitates pathogen entrapment and amplifies thrombo-inflammatory responses. Proteolytic regulators and scavengers (top left panel): Neutrophil elastase proteolytically processes thrombin, releasing C-terminal fragments such as GKY25 and HVF18, which function as biophysical scavengers capable of neutralizing lipopolysaccharide (LPS) and other pro-inflammatory ligands, thereby limiting excessive inflammation. Pathogen-specific response (bottom left panel): *Staphylococcus aureus* α-toxin induces the selective release of cytoplasmic hBD-1 from platelets, triggering NETs formation and establishing an alternative, pathogen-tailored activation pathway for bacterial immobilization and clearance.

Although LL-37 acts as a prototypical broad-spectrum platelet agonist, it does not consistently promote coagulation, which is regulated by both pro-thrombotic accelerators and inhibitory mechanisms that modulate the intravascular response to infection. Key accelerators include human neutrophil α-defensins (HNP 1-3) and platelet factor 4 (PF4). In contrast to the amphipathic helix insertion mechanism of LL-37, HNP-1 employs a structural locking mechanism by directly modulating integrin αIIbβ3 and promoting the precipitation of fibrinogen and thrombospondin-1 into an insoluble, amyloid-like network [29,47,48]. HNP-induced fibrils exhibit distinct biophysical properties compared to canonical fibrin clots; they are mechanically rigid and resistant to fibrinolysis by tPA and plasmin, resulting in a matrix that is optimized for pathogen entrapment rather than reversible hemostasis [29]. PF4 further reinforces this axis by forming tetrameric complexes with polyanions, such as NETs, DNA, and bacterial surfaces [49]. These complexes stabilize the immunothrombotic scaffold against nuclease degradation and, in pathological contexts, serve as antigens for HIT (Heparin-Induced Thrombocytopenia) and VITT (Vaccine-Induced Immune Thrombotic Thrombocytopenia) antibodies [50,51]. By understanding the roles of LL-37, HNP-1, and PF4 as critical nodes in an immune-coagulation feedback loop, we can better appreciate how these divergent actions collectively shape the complex landscape of immunothrombosis [25,52,53]. This intricate feedback mechanism underscores the bidirectional signaling between coagulation factors and innate immunity, highlighting the dynamic interplay that can influence the body’s response to infection and inflammation (Figure 3).

Beyond granular proteins, human β-defensins (hBDs) exhibit distinct compartmentalized functions in thrombo-inflammation. hBD-1, localized within the platelet extragranular cytoplasm, is selectively released in response to bacterial pore-forming toxins such as *S. aureus* α-toxin. This release initiates NETs formation, establishing a pathogen-specific activation pathway that operates independently of classical degranulation [54]. In contrast, hBD-3 is sequestered within platelet-derived extracellular vesicles (p-EVs). Upon platelet activation, these vesicles transport hBD-3 to the endothelium, leading to downregulation of endothelial nitric oxide synthase (eNOS) and upregulation of von Willebrand Factor (vWF), collectively fostering a prothrombotic vascular microenvironment at a distance [55].

Under systemic oxidative stress, the peptide Dermcidin (DCN-2), typically associated with sweat, acts as a potent systemic platelet aggregator. Dermcidin synergizes with ADP to increase thrombosis by more than 500-fold and inhibits endothelial nitric oxide synthesis, thereby promoting a prothrombotic state [56]. To restrict the transition from localized immunothrombosis to systemic disseminated intravascular coagulation, the host response employs molecular brakes embedded within coagulation factors. A paradigm of this regulation is the proteolytic processing of thrombin by neutrophil elastase, which removes thrombin’s procoagulant enzymatic activity, thereby limiting clot propagation, and simultaneously liberates C-terminal host defense peptides, such as HVF18 and GKY25, which possess distinct immunomodulatory properties. These HDPs, like LL-37, bind LPS with high affinity, inducing its aggregation into large complexes. This sequestration physically prevents LPS from engaging the CD14/Toll-like receptor 4 (TLR4) complex on macrophages. Consequently, this process exerts a dual protective role: it facilitates rapid pathogen clearance while simultaneously dampening the systemic cytokine storm, thereby structurally decoupling inflammation from coagulation during sepsis [19,57].

Similarly, Lactoferricin, generated by N-terminal cleavage of lactoferrin, and the arthropod peptide Tachyplesin I function as competitive antagonists. Unlike aggregatory defensins, Lactoferricin competes for the LRP1 receptor, preventing the pro-thrombotic signaling cascades typically triggered by ligand accumulation [58,59]. At the same time, Tachyplesin I penetrates cellular membranes to disrupt PI3K/Akt signaling, thus uncoupling G protein-coupled receptor (GPCR) occupancy from the cytoskeletal reorganization required for aggregation [60]. During the resolution phase, salivary peptides such as Histatin-1 facilitate endothelial cell spreading and angiogenesis, redirecting the physiological focus from clot formation to tissue repair [61]. This intricate interplay demonstrates that the immunothrombotic response is not a singular event driven solely by charge, but rather a coordinated sequence of peptide-receptor interactions that balance pathogen containment with the preservation of vascular patency.

## 4. Platelets as Reservoirs and Responsive Effectors of Host Defense Peptides

Platelet-derived host defense peptides play a crucial role in bridging innate immunity and hemostasis, thereby influencing both protective and pathogenic processes. Contrary to the long-held sponge hypothesis, which attributed the presence of immune mediators in platelets to passive endocytosis from plasma, recent transcriptomic and proteomic analyses have provided new insights. These studies refute the old explanation, demonstrating that platelets actively synthesize immune mediators. This process results in a megakaryocyte-derived repertoire of HDPs that are compartmentalized within subcellular structures and released in response to specific stimuli [22,62].

**Figure 4:**
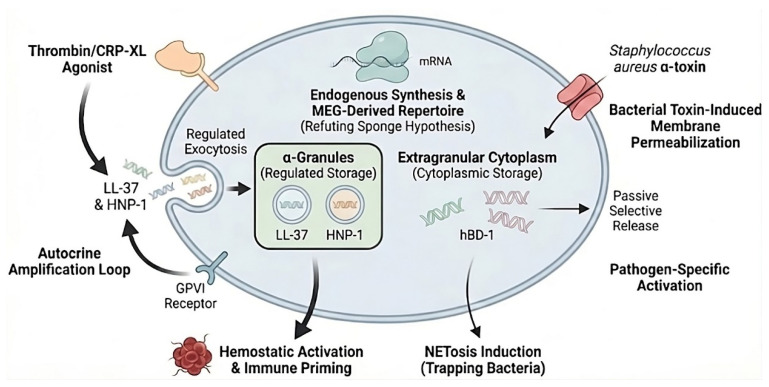
Platelets as active reservoirs and effectors of HDPs. Endogenous mRNA transcription and translation in platelets of HDPs are stored through two distinct pathways: regulated storage, in which LL-37 and human neutrophil peptide-1 (HNP-1) are sequestered in α-granules and released by exocytosis following activation with hemostatic agonists (thrombin or CRP-XL); and cytoplasmic storage, where human β-defensin-1 (hBD-1) resides in the extragranular cytoplasm and is selectively released via toxin-induced membrane permeabilization. Secreted LL-37 subsequently engages GPVI in an autocrine loop, reinforcing platelet immune priming and promoting neutrophil extracellular trap (NET) formation.

The storage of HDPs in human platelets is organized intricately, reflecting distinct functional roles. The cathelicidin LL-37 has been conclusively identified within platelet α-granules. Immunofluorescence and ultrastructural studies demonstrate that LL-37 is synthesized by megakaryocytes, sorted into granules, and transferred to pro-platelets, thereby refuting the notion of exclusive uptake from neutrophils [11]. This structural organization not only underscores platelets’ role in immune defense but also connects it with clinical outcomes, such as the role of LL-37 in conditions like sepsis and thrombosis. Similarly, the presence of α-defensins (HNP-1, -2, -3) in platelets, previously a subject of controversy [54], has been clarified by recent mechanistic studies. Valle-Jiménez et al. demonstrated that human megakaryocytes (MEG-01 models and primary cells) express *DEFA1* mRNA and synthesize the HNP-1 protein, which colocalizes with P-selectin in α-granules [63]. This endogenous synthesis provides platelets with an immediate-response arsenal independent of leukocyte recruitment. In contrast, human beta-defensin 1 (hBD-1) exhibits a distinct localization pattern. Unlike LL-37 and HNP-1, which are stored in granules, hBD-1 is primarily found in the extragranular cytoplasm [54]. The release of HDPs is stimulus-dependent, reflecting specialized adaptations to diverse threats. Hemostatic activation by classical agonists such as thrombin and the collagen-related peptide (CRP-XL) induces regulated exocytosis of alpha-granules, resulting in the secretion of LL-37 and HNP-1 [11,63]. This process integrates antimicrobial defense with hemostatic plug formation. In contrast, the release of cytoplasmic hBD-1 requires a different trigger and is not significantly induced by thrombin. Instead, hBD-1 is predominantly released when bacterial pore-forming toxins, such as Staphylococcus aureus alpha-toxin, compromise platelet integrity [54]. This mechanism functions as a molecular trap, wherein pathogen-induced membrane permeabilization leads to the passive yet selective release of cytoplasmic antimicrobial agents. This nuanced release system parallels leukocyte pattern recognition, enabling platelets to sense specific threats and deploy peptides in a graded, targeted manner, like pathogen discrimination in leukocytes [54,64] (Figure 4).

Beyond their role as carriers, platelets function as active sensors of HDPs. LL-37 serves as a distinct platelet agonist. The secretion of LL-37 creates an autocrine/paracrine amplification loop. As detailed in Section 3, released LL-37 re-engages platelet GPVI receptors, triggering downstream signaling that lowers the activation threshold for secondary agonists [23]. The engagement of a classical hemostatic receptor by an immune peptide underscores the integration of platelets into innate defense, enabling them to sense antimicrobial effectors and amplify thromboinflammatory responses [11].

This interaction primes platelets’ immune systems. At sub-threshold concentrations, HDPs do not cause aggregation but lower the activation threshold for secondary agonists, thereby increasing P-selectin exposure and integrin αIIbβ3 activation [11]. Physiologically, this priming facilitates the rapid formation of platelet-leukocyte aggregates and stabilization of NETs. For instance, platelet-derived hBD-1 is a potent inducer of NETosis, which physically traps bacteria within a prothrombotic matrix [54]. However, this dual function introduces risk: systemic elevation of HDPs, as observed in sepsis or psoriasis, may promote pathological thrombosis (thromboinflammation) by excessively activating the platelet-peptide axis. The therapeutic promise lies in harnessing HDPs for targeted antimicrobial and thrombo-protective interventions. The thrombotic risk involves the possibility of unintentional promotion of pathological thrombosis due to heightened platelet-peptide axis activation. Careful management of these therapeutic potentials and associated thromboinflammatory risks is essential for developing new clinical strategies while minimizing adverse outcomes [16,65].

The regulatory complexity of platelet antimicrobial responses is further illustrated by the human defensin family, which demonstrates distinct storage and release mechanisms. α-Defensins (HNP-1) are synthesized by megakaryocytes, stored in platelet α-granules, and released to enhance fibrinogen binding and stabilize clot architecture by forming a fibrillar network [29,63].

## 5. Exogenous Modulation and Negative Regulation: Lessons from Leukocytes and Evolutionary Biology

While platelets serve as active reservoirs for pro-thrombotic peptides, the hemostatic balance is critically maintained by exogenous modulators derived from the immune microenvironment. A prime example of this transcellular regulation is Lactoferrin. Although it interacts intimately with platelets, this iron-binding glycoprotein is synthesized and released primarily from neutrophil secondary granules, functioning as a “molecular brake” on the platelet-peptide axis. Its antithrombotic functionality depends on the tetrapeptide motif Lys-Arg-Asp-Ser (KRDS), which structurally mimics the Arg-Gly-Asp (RGD) adhesion sequence. By serving as a competitive antagonist at the fibrinogen-binding site of integrin αIIbβ3, the KRDS motif effectively blocks pathological aggregation while preserving initial adhesion [66].

Similarly, Tachyplesin I, a rigid β-hairpin peptide derived from marine arthropods (*Tachypleus tridentatus*), inhibits the Phosphoinositide 3-kinase (PI3K)/Akt signaling pathway explicitly. By penetrating the platelet membrane and blocking Akt phosphorylation at Ser473, Tachyplesin I uncouples surface receptor engagement from the cytoskeletal changes required for clot retraction [60]. However, despite these insights, key unanswered questions remain, including the regulation of peptide sorting in disease states. Addressing these gaps will enhance our understanding and guide future research in developing novel therapeutic strategies.

## 6. Megakaryocytes as Autonomous Immune Sentinels: Transcriptional Regulation and Pathological Adaptability

Recent studies have fundamentally shifted the functional paradigm of the megakaryocyte (MK) from a mere cellular factory to an active immune sentinel capable of endogenously synthesizing HDPs. This challenges the classical view that platelet-associated HDPs are solely derived from plasma endocytosis. Transcriptional profiling via RNA-seq has provided unequivocal evidence of this autonomy; specifically, the robust detection of *DEFA1* mRNA in leukocyte-free platelets and MEG-01 cells demonstrates active transcription rather than passive uptake. Crucially, this process involves sophisticated sorting mechanisms in which *DEFA1* transcripts are not only synthesized but also selectively enriched up to 170-fold in proplatelets compared to stem cells, ensuring that the anucleate progeny is equipped with a genetic template for HNP-1 synthesis [63]. (Figure 4).

This “in-house” production capability is further corroborated by the constitutive expression of *DEFB1* (hBD-1) in MKs—distinct from the uptake-dependent presence of hBD-3—and the detection of *CAMP* (cathelicidin antimicrobial peptide) transcripts—the gene that encodes LL-37—regulated by cAMP signaling pathways essential for megakaryopoiesis [11,54]. This genetic programming results in a precise subcellular geography in which synthesized peptides, such as HNP-1, co-localize with P-selectin in α-granules, synchronizing antimicrobial release with hemostatic activation by thrombin or collagen [63].

**Figure 5:**
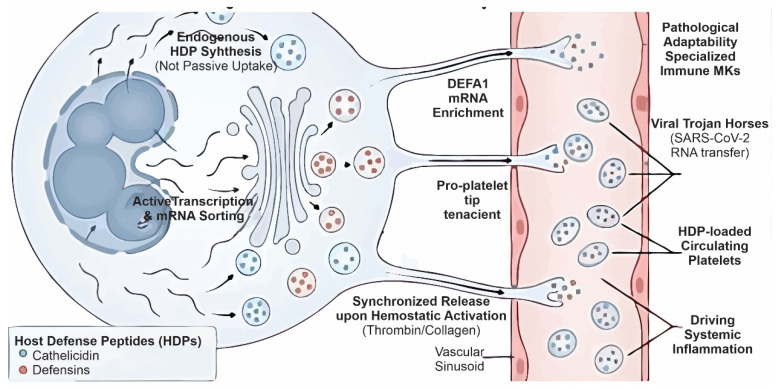
Megakaryocytes as autonomous immune sentinels and their pathological adaptability. The left panel depicts the paradigm shift from passive uptake to active endogenous HDPs synthesis. Megakaryocytes perform regulated transcription and mRNA sorting, enriching defensin transcripts in proplatelet tips for targeted delivery to platelets. The right panel illustrates pathological adaptability: specialized “immune MK” subpopulations can act as viral “Trojan horses” (e.g., in COVID-19), transferring viral RNA to platelets and releasing progeny loaded with HDPs that contribute to systemic inflammation and immunothrombotic complications.

However, single-cell RNA sequencing reveals that this immune competency comes at a cost: specialized “immune MK” subpopulations enriched in myeloid markers. In severe pathologies such as COVID-19, these reprogrammed cells can function as viral “Trojan horses,” transferring SARS-CoV-2 RNA to platelets and driving systemic inflammation. This phenomenon delineates the critical balance between the MK’s protective role in host defense and its potential contribution to mortality [20] (Figure 5).

## 7. Modulation of Vascular Tone and Endothelial Function by Antimicrobial Peptides and Functional Consequences

The vascular endothelium serves as a critical sensory and effector organ, maintaining hemodynamic homeostasis through the delicate balance of vasodilators, such as nitric oxide (NO) and prostacyclin, and vasoconstrictors, notably endothelin-1 (ET-1) [67]. HDPs have emerged as potent, non-canonical modulators of this equilibrium, exhibiting distinct functions based on peptide family and pathological context [68]. Disruption of endothelial homeostasis can result in vascular disorders, including hypertension and atherosclerosis, underscoring the significance of the NO–ET-1 balance in cardiovascular health.

**Figure 6:**
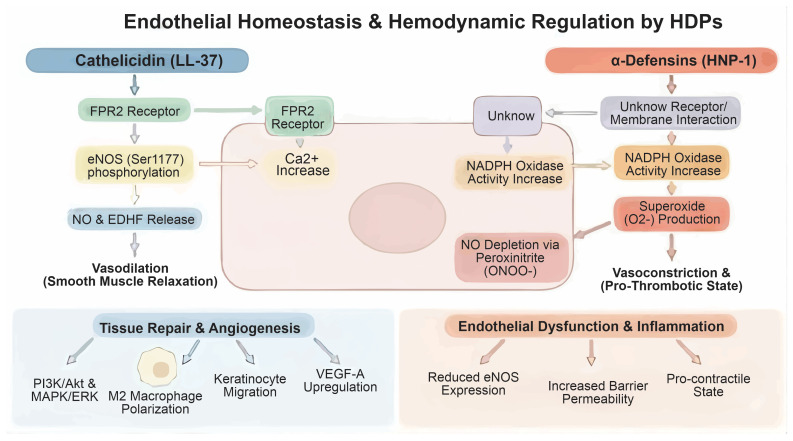
Endothelial homeostasis and hemodynamic regulation by antimicrobial peptides (HDPs). The upper panel illustrates the functional dichotomy of HDPs. LL-37 (cathelicidin) promotes vasodilation via FPR2-mediated Ca^2+^ mobilization and eNOS phosphorylation (Ser1177), leading to nitric oxide (NO) and EDHF release. In contrast, HNP-1 (α-defensin) induces endothelial dysfunction and a pro-thrombotic state by enhancing NADPH oxidase activity and superoxide (O_2_•^−^) production, which depletes NO through peroxynitrite (ONOO^−^) formation. The lower panels depict broader consequences: LL-37 coordinates tissue repair, angiogenesis, and M2 macrophage polarization via PI3K/Akt and MAPK/ERK signaling, whereas HNP-1-driven oxidative stress increases endothelial barrier permeability and inflammation.

LL-37 induces endothelium-dependent vasodilation by activating the FPR2 receptor. LL-37 initiates intracellular calcium mobilization and phosphorylation of endothelial nitric oxide synthase (eNOS) at Ser1177, resulting in rapid NO release. Additionally, LL-37 stimulates the release of endothelium-derived hyperpolarizing factor (EDHF), which, in conjunction with NO, hyperpolarizes smooth muscle cells, particularly within the venous circulation [69] (Figure 6). Beyond hemodynamic regulation, LL-37 modulates endothelial structural integrity. Recent biomechanical studies demonstrate that LL-37 regulates endothelial cell stiffness and barrier permeability [70].

In contrast to LL-37, which induces vasodilation, α-defensins (HNP1-3) promote endothelial dysfunction and vasoconstriction, emphasizing the functional dichotomy among peptide families. Pathologically relevant concentrations of HNP-1 significantly reduce endothelium-dependent vasorelaxation in coronary arteries. This effect is mediated by decreased expression of endothelial nitric oxide synthase (eNOS) mRNA and protein, along with increased NADPH oxidase activity. The resulting elevation in superoxide anions (O_2_•^−^) depletes nitric oxide (NO) by forming peroxynitrite (ONOO^−^), thereby reducing NO bioavailability and predisposing vessels to a pro-contractile and pro-thrombotic state. When the superoxide/NO ratio surpasses a critical threshold, vascular responses shift from vasodilation to vasoconstriction, illustrating the delicate balance between oxidative and nitrosative stress that governs vascular function. This oxidative stress impairs endothelial function and elevates thrombotic risk, potentially leading to severe cardiovascular events such as acute coronary syndrome [71].

LL-37 acts as a potent inducer of both physiological and pathological angiogenesis. It promotes endothelial cell proliferation, migration, and tubulogenesis by activating FPR2 and recruiting monocytes and neutrophils [72,73]. LL-37 can also transactivate the epidermal growth factor receptor (EGFR) and upregulate vascular endothelial growth factor A (VEGF-A) expression in keratinocytes and endothelial cells, creating a feed-forward loop that accelerates vascular growth during tissue repair [74].

HDPs can exert both pro-angiogenic and anti-angiogenic effects, depending on their specific properties. For example, dermaseptins, particularly dermaseptin-B2 and its analogs, exhibit strong anti-angiogenic activity [75]. Although initially identified in amphibian skin, their mechanisms of action on mammalian endothelium offer valuable insights into HDPs’ biology. These peptides inhibit tumor angiogenesis by disrupting endothelial cell cytoskeletal integrity, suppressing proliferation, and blocking VEGF signaling, thereby depriving neoplastic tissues of their blood supply. The balance between pro- and anti-angiogenic effects is likely determined by receptor affinity, such as FPR2 compared to heparan sulfate proteoglycans, and by the specific signaling interactions induced by the peptide’s secondary structure [74]. HDPs also regulate vascular tone throughout the wound-healing continuum, including hemostasis, inflammation, proliferation, and remodeling. During hemostasis and inflammation, rapid release of HNP1-3 and LL-37 from degranulating neutrophils contributes to the formation of a thrombo-inflammatory barrier. LL-37, the neutrophil granule, further promotes coagulation, stabilizes NETs, and recruits monocytes and neutrophils to the injury site via FPR2 and CXCR2 chemokine receptors [73].

During the proliferative and re-endothelialization phases, LL-37 facilitates tissue regeneration by inducing cellular migration via EGFR activation and Snail transcription factor induction, a mechanism that is fundamental for restoring the functional vascular lining. This process is complemented by LL-37-driven macrophage polarization toward an M2-like repair phenotype, which suppresses acute inflammation and secretes essential growth factors required for matrix deposition and remodeling [76,77].

This multiphasic regulation underscores the dual role of HDPs as antimicrobial agents and as critical growth factors that coordinate immune responses with tissue regeneration. Further research is required to determine whether timed delivery of LL-37 can optimize the transition to M2 macrophages without inducing excessive fibrosis, a key consideration in wound healing.

## 8. Emerging Therapeutic Strategies and Prospective Research Directions

The evolution of HDPs from simple antibiotics to pleiotropic regulators of immunothrombosis has redefined their therapeutic potential (Table 1). This section evaluates current clinical candidates, advanced bioengineering strategies like protease-resistant peptidomimetics, and biomaterials. Finally, we examine how established therapies, including statins and vitamin D, modulate endogenous HDP expression to achieve vascular protection while mitigating thromboinflammatory risks [77,78,79,80].

Despite progress, the clinical application of native peptides such as LL-37 and α-defensins is severely constrained by rapid proteolytic degradation in vivo and the significant risk of off-target cytotoxicity, currently estimated at approximately 20–30% in relevant therapeutic contexts. An alternative strategy to reduce off-target cytotoxicity is to exploit the natural synergy between HDPs. Recent biophysical studies using Fluorescence Recovery After Photobleaching (FRAP) on model mammalian membranes (POPC bilayers) have demonstrated a “double-cooperative” effect. While LL-37 alone disrupts lipid bilayer integrity at therapeutic concentrations (9 μM), the co-administration of human defensins (HNP-1, HNP-3, or hBD-1) in a 1:1 molar ratio effectively rescues membrane stability and prevents lysis [81].

**Table 1:** Human host defense peptides as regulators of hemostasis and vascular biology. Overview of the human host defense peptides (HDPs) discussed in this review, summarizing their primary cellular sources, key structural and receptor features, major hemostatic and vascular effects, and their pathological or therapeutic implications, designed to harmonize nomenclature, facilitate comparison across peptide families, and improve readability by integrating canonical antimicrobial functions with emerging non-canonical roles in hemostasis, thrombosis, and vascular biology.

HDP/Primary Source (Subcellular Localization)	Structure/Main Receptors/Targets	Hemostatic and Vascular Effects	Pathological/Therapeutic Implications
LL-37—neutrophils, epithelium; synthesized by megakaryocytes; platelet α-granules [11,63].	α-helical, oligomerizes; FPR2/ALX, GPVI, Src/Akt, Syk → PLCγ2, EGFR [35,36,37,38,72,82].	Platelet activation (sensitizes), Ca^2+^ mobilization; ↑ eNOS/EDHF; pro-angiogenic; NET stabilization [11,16,24,72].	Drives immunothrombosis in sepsis; wound-healing candidates (ropocamptide/KR-12) with safety trade-offs [11,83].
α-Defensins (HNP-1–3)—neutrophil azurophilic granules; platelet α-granules [63,84]	Cysteine-stabilized β-sheets; bind αIIbβ3, fibrinogen, thrombospondin [29,48].	Forms rigid, amyloid-like fibrin networks; resistant to fibrinolysis; promotes endothelial oxidative stress (↓ NO) [48,71].	Stabilize pathological thrombi; implicated in plaque instability; modulation reduces amyloid-clot effects [48,85].
β-Defensin-1 (hBD-1)—epithelium; platelet extragranular cytoplasm (released by toxins) [54].	Cationic, membrane-active [54].	Triggered release → NETosis; local antimicrobial trap that scaffolds prothrombotic NETs [54].	Rapid pathogen containment but potentially localized thrombo-inflammation [54].
β-Defensin-3 (hBD-3)—epithelium; platelet extracellular vesicles (p-EVs) [55].	Cationic; EV-packaging for distal delivery [55].	EV-mediated ↓ eNOS and ↑ vWF in endothelium → distal pro-coagulant signaling [55].	Propagates endothelial dysfunction at a distance; target to block EV effects [55].
Lactoferrin/Lactoferricin—neutrophil secondary granules; secretions/plasma [12,58].	Iron-binding glycoprotein; lactoferricin = cationic fragment; interacts with LRP1 and integrin pathways; affects AMPK/mTOR [12,13,58].	Inhibits platelet aggregation (competitor); promotes macrophage autophagy; preserves endothelial integrity [12,13].	Antithrombotic/vasoprotective; fragment templates for anticoagulant therapeutics [12,13].
PF4 (platelet factor 4)—platelet α-granule release; thrombi/NETs enriched [49].	Cationic chemokine that tetramerizes and binds polyanions (DNA/heparin) [49].	Stabilizes NET/thrombus scaffold; protects from nuclease degradation [49,50,51].	Central in HIT/VITT; immune-mediated thrombosis risk; clinically relevant target [49,50,51].
Dermcidin (DCN-2)—sweat glands; systemic under stress [56].	Secreted sweat peptide [56].	Potent platelet aggregator; synergizes with ADP; inhibits endothelial NO → prothrombotic [56].	Links stress to thrombosis (MI contexts) [56].
Thrombin-C peptides (HVF18, GKY25)—thrombin proteolysis (neutrophil elastase) [19,57].	Short cationic C-terminal fragments; high LPS affinity [57].	Sequester LPS → dampen TLR4 signaling; LPS scavenging without procoagulant activity [19,57].	Endogenous brakes in sepsis; templates for LPS-scavenging therapeutics [19,57].
Histatin-1—saliva (salivary glands) [61].	Histidine-rich, adhesive [61].	Promotes endothelial adhesion, migration, and angiogenesis → repair phase [61].	Candidate for biomaterials to assist vascular repair [61].
KR-12/engineered HDP analogs (ropocamptide, Brilacidin, SAAP-148)—LL-37 fragments/mimetics in devices and trials [86,87,88,89,90].	Short amphipathic fragments or stabilized mimetics (protease-resistant) [86,87,88,89,90].	Local hemostasis + antimicrobial action; promote re-epithelialization; aim to reduce systemic toxicity [86,87,88,89,90].	Promising for wounds and local hemostasis; systemic stability/toxicity remain barriers [86,87,88,89,90].

Abbreviations: HDPs, host defense peptides; LL-37, human cathelicidin antimicrobial peptide; HNPs, human neutrophil peptides (α-defensins 1–3); hBD-1 and hBD-3, human β-defensin 1 and 3; PF4, platelet factor 4; NETs, neutrophil extracellular traps; p-EVs, platelet-derived extracellular vesicles; FPR2/ALX, formyl peptide receptor 2/lipoxin A4 receptor; GPVI, glycoprotein VI; PLCγ2, phospholipase C gamma 2; eNOS, endothelial nitric oxide synthase; EDHF, endothelium-derived hyperpolarizing factor; vWF, von Willebrand factor; Akt, protein kinase B; AMPK, AMP-activated protein kinase; mTOR, mechanistic target of rapamycin; LRP1, low-density lipoprotein receptor-related protein 1; TLR4, Toll-like receptor 4; HIT, heparin-induced thrombocytopenia; VITT, vaccine-induced immune thrombotic thrombocytopenia.

### 8.1. Clinical Translation and Therapeutic Potential of HDPs and Engineered Mimetics

Ropocamptide (LL-37), a synthetic analogue, addresses local peptide deficiencies in chronic venous leg ulcers (VLUs). In the Phase IIb HEAL trial (n = 148), although the primary endpoint was not met in the overall population, a subgroup analysis of large refractory ulcers (>10 cm^2^) showed a significant difference in complete healing (28.8% vs. 8.1% with placebo; *p* < 0.05). Topically applied ropocamptide was well tolerated, validating the safety of HDP-based therapies in vascularly compromised beds, though its translation is currently stalled by financial rather than scientific hurdles [91].

A non-peptide defensin-mimetic (arylamide foldamer), Brilacidin, leverages HDP amphipathicity within a backbone resistant to proteolysis. Brilacidin has demonstrated potent antiviral activity against SARS-CoV-2 by blocking viral entry and disrupting viral integrity. Furthermore, in Phase II (ClinicalTrials.gov ID NCT02324335) trials for oral mucositis, it significantly reduced the incidence of severe ulceration [92].

In Phase IIb trials for Acute Bacterial Skin Infections (ABSSSI), a single intravenous dose matched the efficacy of a 7-day Daptomycin regimen, advancing antibiotic stewardship. Its pleiotropic profile includes blocking viral entry via heparan sulfate proteoglycans (SI = 426) and mitigating severe oral mucositis in oncology patients by attenuating TNF-α and IL-1β release [83,93].

Peptide Peceleganan (PL-5) is one of the HDPs screened from hundreds of candidates in our rationally designed library, a chemically synthesized α-helical HDP containing 26 amino acid residues. PL-5 spray is the first topical HDP spray agent specifically developed for skin wound infections. In preclinical studies, as compared with conventional antibiotics, PL-5 showed a stronger and broader spectrum of antibacterial activities against both Gram-positive and Gram-negative bacteria, which was analyzed in a 2024 Phase III trial (n = 570), establishing Peceleganan’s superiority over silver sulfadiazine in infected wounds (90.4% vs. 78.7% efficacy; *p* < 0.001). Despite high clinical success, it showed lower bacterial clearance at day 5 (16.5% vs. 30.7%), suggesting its benefit is driven by immunomodulation and repair acceleration rather than exhaustive sterilization. Negligible systemic absorption ensures a high safety profile, avoiding the nephrotoxicity associated with traditional polypeptide antibiotics [94,95].

LL-37-derived peptides P60.4Ac, OP-145, and SAAP-148 were developed for the treatment of chronic suppurative otitis media; these HDPs highlight the importance of sequence optimization. Peptide P60.4Ac is an interesting candidate because it also displays anti-inflammatory activities, including lipopolysaccharide-neutralizing activity, and has been found to be safe and well-tolerated (0.5 mg of peptide/mL). It has been selected for the subsequent phase IIa study. While OP-145 established safety in Phase I/II trials, stability challenges led to the development of SAAP-148. This optimized derivative exhibits superior stability and bactericidal activity against multidrug-resistant (MDR) biofilms and persister cells, marking a successful transition from proof-of-concept to a viable clinical candidate [86,96].

### 8.2. Bioactive Interfaces

The application of HDPs goes beyond soluble drugs to functionalized biomaterials, where they serve as bioactive coatings to prevent device-associated thrombosis and infection.

For traumatic injury and surgical hemostasis, microporous starch sponges have been functionalized with KR-12, the smallest antimicrobial fragment of human LL-37 (residues 18–29) [87]. KR-12 was covalently attached to starch sponges (KR-Sps) via a thiol-ene photo-click reaction. This method ensures a high loading density and orients the peptide, preserving its bioactive cationic face. The functionalized sponges work through a dual mechanism: rapid hemostasis via water absorption and concentration of clotting factors, followed by sustained antimicrobial activity against MRSA and E. coli for up to 5 days. In vivo models showed that KR-12 sponges reduced blood loss, decreased inflammation, and promoted keratinocyte proliferation, supporting the use of HDPs in wound care devices that address both bleeding and infection risks [87,88,89,90].

### 8.3. Diagnostic and Prognostic Utility

HDPs function as sensitive biomarkers for systemic inflammation and vascular instability. In sepsis, particularly in neonates, plasma LL-37 levels rise sharply and serve as critical diagnostic indicators for timely intervention. In severe adult sepsis, LL-37 levels inversely correlate with platelet counts, reflecting peptide consumption during widespread immunothrombosis. Conversely, elevated HNP 1–3 concentrations in atherosclerotic plaques independently correlate with major adverse cardiovascular events (MACE) and mortality, underscoring their role in plaque instability and in inhibiting fibrinolysis [29,84,97].

### 8.4. Pharmacological Modulation: Repurposing Established Drugs

Beyond their classical lipid-lowering effects, statins (e.g., simvastatin, atorvastatin) exert pleiotropic immunomodulatory actions by inducing LL-37 (CAMP) expression through a mevalonate-independent pathway. This induction is mediated by the inhibition of histone deacetylases (HDACs), which upregulates CYP27B1 transcription to facilitate the conversion of inactive 25-hydroxyvitamin D3 into active 1,25-dihydroxyvitamin D3. The subsequent binding of 1,25(OH)2D3 to the vitamin D receptor (VDR) triggers CAMP transcription, providing a molecular basis for the synergistic antimicrobial activity observed during combined statin and vitamin D supplementation [82,98].

Similarly, the HDAC inhibitor phenylbutyrate (PBA) is a potent inducer of LL-37, promoting intracellular killing of Mycobacterium tuberculosis by activating P2RX7 receptor-dependent autophagy. This signaling axis, involving cytosolic Ca2+ mobilization and activation of the AMPK/PI3K pathway, positions PBA and vitamin D as viable host-directed therapies to bolster endogenous defenses against antibiotic-resistant pathogens [99].

Furthermore, the anti-inflammatory alkaloid colchicine—recently approved for cardiovascular risk reduction—modulates HNP 1–3 levels by stabilizing microtubules and inhibiting neutrophil degranulation. By attenuating the release of HNP 1–3, colchicine reduces the formation of amyloid-like fibrin clots and enhances atherosclerotic plaque stability, offering a clear mechanistic rationale for its cardioprotective benefits in patients with coronary artery disease [85].

## 9. Conclusions

The convergence of innate immunity and hemostasis defines the pleiotropic nature of HDPs. This review establishes that these peptides are not merely passive antibiotics but sophisticated signaling molecules that orchestrate the “immunothrombotic” response. A critical advance highlighted herein is the recognition of the megakaryocyte-platelet axis as an autonomous producer of immune effectors, refuting the historical “sponge hypothesis.” The structural plasticity of peptides like LL-37 allows them to function as molecular switches—driving platelet activation via GPVI, modulating vascular tone, and stabilizing fibrin scaffolds—depending on the physiological context and local concentration. Translation of these insights into precision medicine requires navigating the fine balance between therapeutic efficacy and pathological thromboinflammation. Future research must prioritize clarifying the role of adaptive thrombopoiesis during systemic infection and defining the molecular “barcodes” that dictate receptor selectivity to engineer safer peptidomimetics. Additionally, integrating HDPs into advanced wound-care biomaterials represents a promising translational frontier. However, it is crucial to consider the pharmacokinetic challenges associated with HDP-loaded biomaterials, particularly their absorption and clearance profiles, which may influence thrombo-inflammatory risk. Acknowledging these hurdles is essential for a balanced precision-medicine outlook. Ultimately, deciphering the regulatory logic of HDPs will be pivotal in developing targeted interventions that exploit their dual protective roles while minimizing collateral vascular damage in complex clinical settings.

## Data Availability

No new data were created or analyzed in this study.
